# AI-based prediction of recurrence after carbon ion radiotherapy for early stage non-small cell lung cancer

**DOI:** 10.1371/journal.pone.0342481

**Published:** 2026-02-10

**Authors:** Yuhei Miyasaka, Hanae Yoshida, Naoko Okano, Hirofumi Shimada, Nobuteru Kubo, Hidemasa Kawamura, Tatsuya Ohno

**Affiliations:** 1 Gunma University Heavy Ion Medical Center, Maebashi, Japan; 2 Department of Radiation Oncology, Gunma University Graduate School of Medicine, Maebashi, Japan; 3 Gunma University Advanced Particle Beam Medical Science Joint Research Course (Hitachi, Ltd.), Maebashi, Japan; 4 Healthcare Innovation Center, Hitachi, Ltd., Tokyo, Japan; Kimura Hospital, JAPAN

## Abstract

Lung cancer is a leading cause of cancer-related deaths. Carbon ion radiotherapy (CIRT) is a treatment modality for patients with inoperable conditions or who decline surgery, but there is room for research to identify patients at high risk of recurrence. The use of artificial intelligence (AI)-based predictive models in healthcare is growing, yet their application in predicting outcomes after CIRT in NSCLC remains unexplored. This study developed an AI prediction model using clinical and imaging data to identify patients at high risk of recurrence after CIRT for early stage NSCLC. Patients with untreated early stage peripheral NSCLC undergoing CIRT between June 2010 and December 2020 were included. Simulated computed tomography (CT) images and clinical data were used to develop a model to predict recurrence within 2 years of CIRT. The model was tested using 5-fold cross-validation and evaluated using receiver operating characteristic (ROC) analysis. The study involved 124 patients. Two-year overall survival, local control, and progression-free survival rates stood at 90.8%, 91.0%, and 69.4%, respectively. The three-axis plane method for CT image input was more predictive than the three-transverse plane or 3D methods. Our AI-based model using CT images and clinical data predicted recurrence within 2 years of CIRT with a median area under the ROC curve of 0.762. Gradient-weighted class activation mapping enhanced model interpretability. The multimodal AI-based model identifies early stage NSCLC patients at high recurrence risk after CIRT although external validation is required for its generalizability and robustness.

## Introduction

Lung cancer is a leading cause of cancer-related deaths worldwide, accounting for 21% of the estimated cancer deaths in the US by 2022 [1]. Approximately 80% of lung cancer cases are non-small cell lung cancer (NSCLC). The standard treatment for early stage NSCLC is lobectomy with mediastinal lymph node resection or segmentectomy. Radiotherapy, such as stereotactic body radiotherapy (SBRT), is indicated for patients with inoperable disease or those who refuse surgery [2]. Carbon ion radiotherapy (CIRT), characterised by a steeper dose distribution and greater biological effectiveness than X-ray radiotherapy [3], is another treatment option for early stage NSCLC. Several clinical studies have shown favourable outcomes [4–10], even in patients with interstitial lung disease [11–13]. Furthermore, although there was no randomized clinical trial comparing CIRT and other modalities, a previous study suggested that CIRT may offer a more favourable prognosis than SBRT in a historical cohort setting [14]. However, evidence shows that the progression-free survival (PFS) rate at 3 years after CIRT is approximately 60% [8,14], underscoring the need to identify high-risk populations.

Recently, the use of artificial intelligence (AI)-based prediction models in healthcare has rapidly increased [15]. This trend extends to radiotherapy for NSCLC [16–19], with studies showing a favourable predictive performance for SBRT [17–19]. Nemoto et al. reported a neural network model using clinical variables to predict cancer progression within 5 years after SBRT [18]. Furthermore, Zheng et al. developed an AI-based model utilising pretreatment CT images and clinical variables to predict overall survival within 2 years of radiotherapy [19]. To date, no AI-based predictions incorporating CT images have been reported for patients with early stage NSCLC treated with CIRT. Multimodal models can provide more accurate predictions of patient prognosis. Thus, we developed and tested a multimodal AI-based prediction model that can be helpful in identifying high-risk populations.

## Materials and methods

### Study design

The present study was designed and conducted according to the principles outlined in the Declaration of Helsinki and the Ethical Guidelines for Medical and Health Research Involving Human Subjects. The Gunma University Hospital Clinical Research Review Board approved this study [IRB2021-056(1947)]. The need for written informed consent was waived because of the historical and observational nature of this study, but all the participants or their relatives had the opportunity to opt-out.

We identified a series of patients who met the following criteria: (i) had pathologically or clinically diagnosed and untreated NSCLC, (ii) had clinical T1a–2bN0M0 (Union for International Cancer Control [UICC], 8th edition), (iii) had peripheral disease, (iv) were treated with CIRT at Gunma University Heavy Ion Medical Center between June 2010 and December 2020, and (v) were followed up for more than 2 years since the initiation of CIRT. Patients with multifocal lung cancer or lung cancer treated within 3 years were excluded. Information of individual participants were identified. Data were accessed between 9 December 2022–1 July 2023.

Simulation CT images with two body positions and clinical data were obtained from all participants and used to develop and test AI-based prediction models for any recurrence or metastasis within 2 years after CIRT.

### Treatment procedures

The following examinations were performed before CIRT: blood cell and biochemical analyses, pulmonary function tests, CT of the thorax and abdomen, whole-brain magnetic resonance imaging or CT, and, in some cases, 18F‐fluorodeoxyglucose positron emission tomography (18F-FDG-PET). Based on these examinations, the appropriateness of CIRT was determined by the Institutional Cancer Board, which included pulmonologists, respiratory surgeons, radiation oncologists, and diagnostic radiologists. Details of the CIRT procedures have been reported elsewhere [8] and are shown in **S1 File**.

After treatment, the patients were followed up with blood tests, including measurement of tumour markers, and CT scans or chest radiographs. In principle, examinations were performed every 1–3 months for the first 3 years, and every 3–6 months thereafter. For patients who refused regular follow-ups at our hospital, their clinical course was obtained via telephone, letters, and patient referral documents.

### Development and testing of predictive models

**Fig 1:**
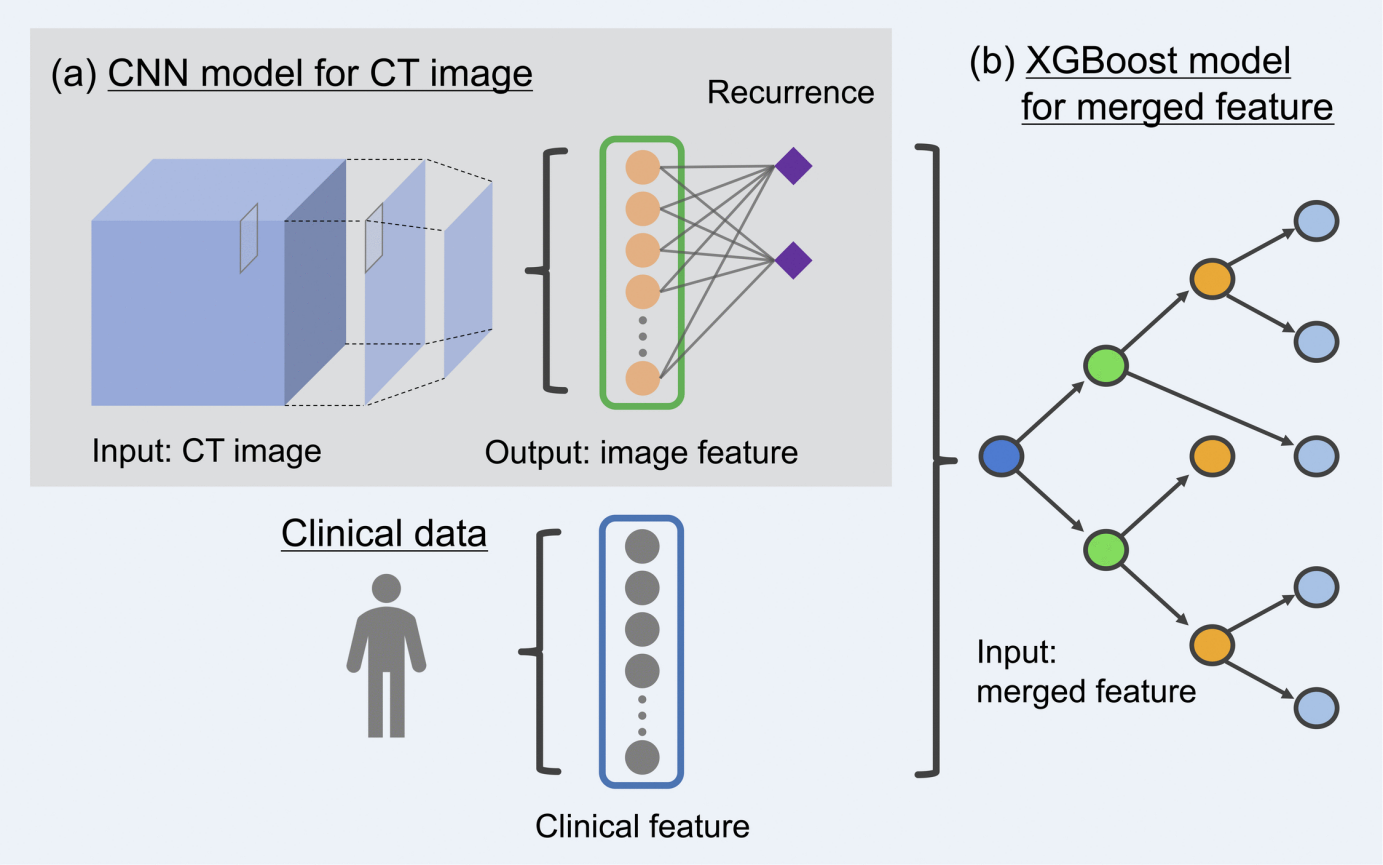
Illustration of development of AI-based models. Image features were obtained from simulation CT images **(a)**. The image and clinical features were used to develop an integrated model with XGBoost **(b)**.

An outline of the predictive model is shown in **Fig 1**. A machine learning framework, PyTorch, was used to develop and test the prediction models [20].

The prediction models included CT images represented in three transverse planes, three axis planes, and three-dimensional (3D) images. To decide which model to include in the integrated model, we performed 5-fold cross-validation. The cohort was randomly divided into five equal groups; each group consisted of 24 or 25 patients, in which three groups were used as the training set, one group as the validation set, and the remaining group as the test set. To avoid obtaining contingent results, we repeated the 5-fold cross-validation five times and evaluated the median values. In addition, focus maps showing where the deep learning model was focused were generated using gradient-weighted class activation mapping (Grad-CAM) [21].

The integrated predictive model included the following clinical factors: age at the initiation of CIRT, sex, Eastern Cooperative Oncology Group performance status score, operability, Brinkman index, T stage (UICC, 8th edition), maximum SUV of 18F-FDG-PET binarized with a threshold of 2.5, and the prescribed dose. These factors and the aforementioned image features were used to develop a prediction model with extreme gradient boosting (XGBoost) [22]. XGBoost incorporates regularization, tree depth control, subsampling, and learning-rate shrinkage, which jointly mitigate overfitting and stabilize performance. Additionally, XGBoost can construct a decision tree by first generating a decision tree (learner) using only data with no missing values, and then assigning the data with missing values to the side with the best score for each segmentation. This feature enables the generation of a prediction model without the imputation of missing values, even for data containing missing values [22]. Hyperparameter tuning for model development was performed using Optuna (**S1 Table**) [23]. A calibration plot was generated to assess the agreement between predicted probabilities and observed outcomes.

### Statistical analyses

Overall survival (OS), PFS, and local control (LC) rates were estimated from the first day of irradiation using the Kaplan–Meier method. Receiver operating characteristic (ROC) analysis was performed to evaluate the performance of the prediction models and areas under the ROC curves (AUCs). The AUC and 95% confidence interval (CI) were calculated using the DeLong method. All analyses and visualisations were performed using R 3.6.2 (R Core Team, Vienna, Austria) [24].

## Results

### Clinical outcomes

**Fig 2:**
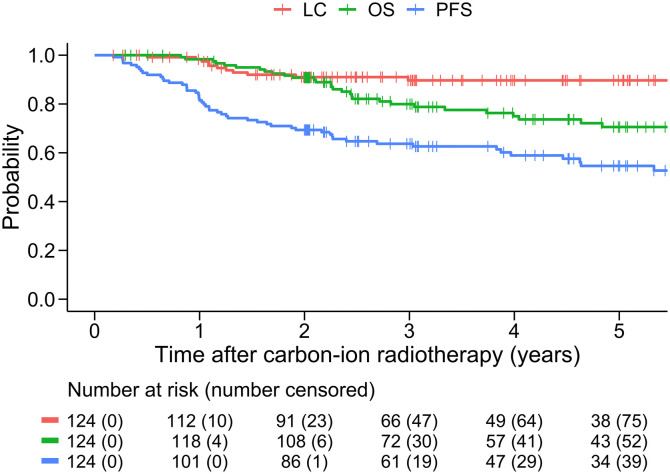
Kaplan-Meir curves for the entire cohort (n = 124). Red, green, and blue lines show LC, OS, and PFS, respectively. Abbreviations: LC, local control; OS, overall survival; PFS, progression-free survival.

A total of 124 patients met the inclusion criteria and were included in the study. The patient characteristics are summarised in **Table 1**. The median follow-up period was 42.3 months (range, 3.5–137). The OS rates were 90.8% (95% CI, 85.7–96.1) at 2 years and 70.6% (95% CI, 61.8–80.6) at 5 years. LC was 91.0% (95% CI, 85.8–96.5) at 2 years and 89.7% (95% CI, 84.0–95.7) at 5 years. PFS was 69.4% (95% CI, 61.7–78.0) at 2 years and 54.6% (95% CI, 45.7–65.2) at 5 years (**Fig 2**). There were 34 disease progression events within 2 years of CIRT, including 10 local recurrences, 17 regional lymph node metastases, and 23 distant metastases. Grades 2 and 3 radiation pneumonitis were observed in two (1.6%) patients. No patient developed grade 4 or higher adverse events.

**Table 1:** Patient characteristics.

Characteristic	N = 124^1^
Sex	
Male	86 (69%)
Female	38 (31%)
Age at treatment	76 (68–82)
ECOG performance status score	
0	45 (36%)
1	70 (56%)
2	9 (7.3%)
Pathological diagnosis	
Adenocarcinoma	52 (42%)
Squamous cell carcinoma	24 (19%)
Unknown (clinical diagnosis)	48 (39%)
Brinkman Index	660 (0–1155)
Unknown	1
Comorbidity	
Chronic obstructive pulmonary disease	51 (41%)
Interstitial pneumonia	7 (5.6%)
History of lung cancer	22 (18%)
Other respiratory disease	25 (20%)
Home oxygen therapy	6 (4.8%)
Diabetes mellitus	26 (21%)
Collagen disease	3 (2.4%)
Cerebrovascular disease	9 (7.3%)
Cardiovascular disease	44 (35%)
Operability	
Operable	77 (62%)
Inoperable	46 (37%)
Unknown	1 (0.8%)
T stage (UICC 8th)	
1 mi	3 (2.4%)
1a	9 (7.3%)
1b	50 (40%)
1c	29 (23%)
2a	26 (21%)
2b	7 (5.6%)
Disease location	
Left lung	55 (44%)
Right lung	69 (56%)
Upper lobe	68 (55%)
Middle lobe	7 (5.6%)
Lower lobe	49 (40%)
Max SUV value	
< 2.5	37 (30%)
≥ 2.5	87 (70%)
Treatment year	
2010–2015	67 (54.0%)
2016–2020	57 (46.0%)
Dose fraction	
52.8 Gy (RBE) in 4 fractions	41 (33%)
60.0 Gy (RBE) in 4 fractions	83 (67%)

^1^Statistics presented: N (%); median (IQR).

ECOG, Eastern Cooperative Oncology Group; Gy (RBE), Grey (Relative Biological Effectiveness); IQR, Interquartile Range; Max SUV, Maximum Standard Uptake Value.

T stage; (UICC 8th), Tumour stage (Union for International Cancer Control, 8th Edition); UICC, Union for International Cancer Control.

### Development of the prediction model

The details of the convolution neural networks are shown in **S1 Fig** and **S2 Table**. In the development of models using CT images, we tested the following three models: the three-transverse plane model, three-axis plane model, and the 3D image model. The 5-fold cross-validation, repeated five times, demonstrated that the median AUC values in the test sets were 0.714 in the three-transverse plane model, 0.746 in the three-axis plane model, and 0.706 in the 3D image model (**S2 Fig**).

**Fig 3:**
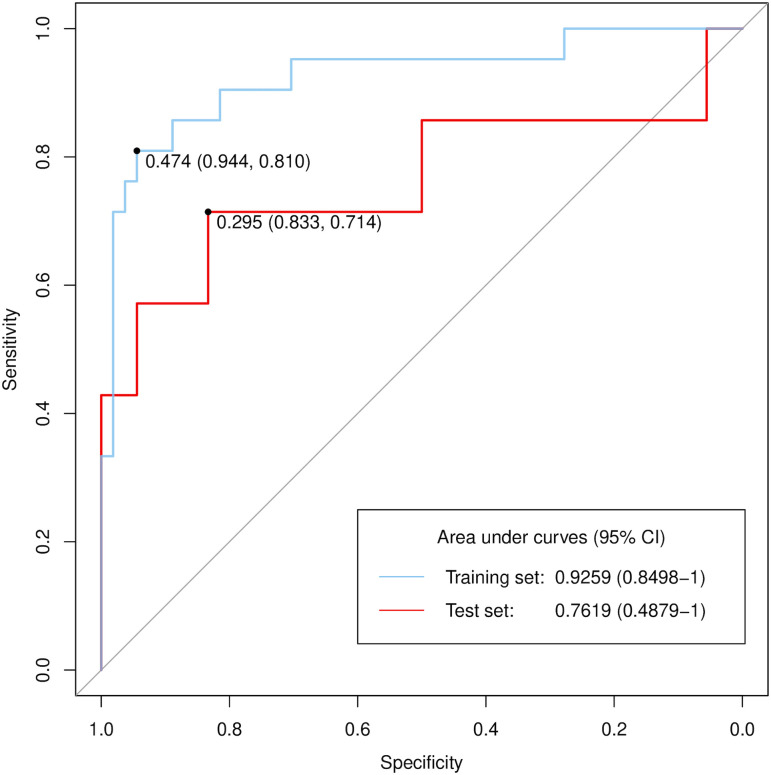
ROC curves of the integrated model using clinical data and CT images by three axes planes. CI, confidence interval.

**Fig 4:**
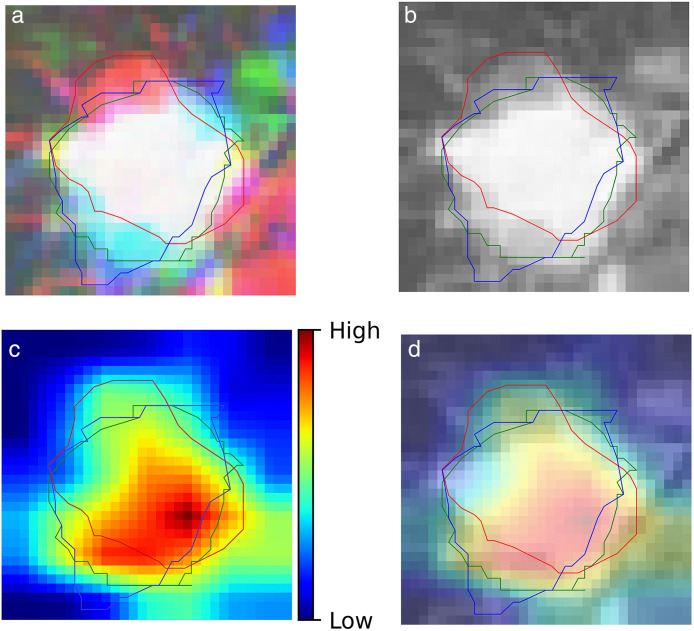
Input and Grad-CAM images. Input images in **(a)** RGB and (b) greyscale. **(c)** Grad-CAM image and (d) the fusional image of (b) with **(c)**. Lines indicate contours of gross target volume in each plane.

Consequently, we incorporated a model based on three-axis planes into the integrated model. The integrated model achieved a median AUC of 0.762 (95% CI, 0.487–1) (**Fig 3**). The calibration curve showed a moderate deviation from the ideal line, with a tendency to underestimate risk at lower predicted values and overestimate risk at higher predicted values (**S3 Fig**). The focus maps were obtained using Grad-CAM (**Fig 4**).

## Discussion

In this study, we developed and tested an AI-based recurrence prediction model in patients with newly diagnosed early stage peripheral NSCLC treated with CIRT at 52.8 or 60.0 Gy (RBE) in four fractions. With respect to the CT input methods, the three-axis plane model was slightly better than the three-transverse plane and 3D input models. Furthermore, the integrated model using CT images and clinical data predicted recurrence within 2 years, with a median AUC of 0.762. To our knowledge, this is the first study to report AI-based recurrence prediction models for prognosis after CIRT in patients with early stage NSCLC.

Several recent studies have reported AI-based prediction models for outcomes after SBRT [16,18,19]. Nemoto et al. developed and tested a prediction model for cancer progression in the first 5 years after SBRT for Tis–4N0M0 NSCLC using clinical data and achieved an AUC of 0.72 in the internal validation cohort and 0.70 in the external validation cohort [18]. Zheng et al. reported an AI-based model for predicting 2-year OS after SBRT in patients with stage I-IIIA NSCLC. Their model using pretreatment CT images and clinical features, including age, sex, T stage, clinical stage, and BED10, achieved a median AUC of 0.76 in the internal validation cohort and 0.64 in the external cohort.

Our AI-based models aimed to predict any recurrence within two years after CIRT, intentionally excluding the impact of other treatment modalities, such as systemic therapy for recurrent disease. This approach may be beneficial for patient decision-making. Our model’s performance, with a median AUC of 0.762, was comparable despite having a smaller and more selective population. In addition, the calibration curve confirmed the validity of our model. We believe that, in the future, it would be beneficial to develop a predictive model that can recommend the most appropriate treatment option.

Employing a focus map to highlight the significant features is useful for understanding AI-based prediction models. We applied the widely used Grad-CAM technique to create a focus map. Grad-CAM generates a localisation map by leveraging the gradients associated with a target concept as they propagate back to the last convolutional layer, highlighting the image areas critical for predicting the concept [21]. **Fig 4** shows that the focus map identified hotspots within a solid nodule. Although there is not quantitative analysis, it were observed in the most of cases we evaluated, supporting the rationality of the model. Interpretability becomes crucial, especially when an AI-based model is developed from a cohort of a limited size.

### Limitations

This study had several limitations. First, our cohort was single-institutional and relatively small, and the robustness of the performance of our model was not sufficiently evaluated because this study did not include an external cohort. It should be noted that model performance generally tends to be lower in an external cohort. Additionally, the relatively small dataset would partially cause the miscalibration. Future studies are warranted to externally test our model. Second, as we included patients over a decade, advances in medication and supportive care would have influence on outcomes. Third, there may be room for improvement in model performance because our dataset did not include information of driver mutations and programmed cell death ligand 1(PD-L1) expression. Fourth, our study included approximately 39% of patients without a pathological diagnosis. While it means our predictive model is applicable for these patients, the impact of pathological diagnosis on recurrence could not be sufficiently considered. Fifth, as our model predicts any recurrence, factors specifically associated with LC, lymph node metastasis, or distant metastasis are unknown. Finally, it is unknown whether our model can be utilised for patients with centrally located, multifocal, advanced, and previously treated NSCLC, as our study did not include such patients.

## Conclusion

We developed and tested a multimodal AI-based model that predicted recurrence within 2 years of carbon ion radiotherapy in newly diagnosed, early stage, peripheral NSCLC patients in a single institutional cohort. Although external validation is required, the predictive performance of our model is feasible.

## Supporting information

S1 Fig Schema of convolutional neural network. (PDF)

S2 Fig ROC curves of CNN models using CT images in the form of (A) three transverse planes, (B) three axes planes, and (C) three-dimensional images. (PDF)

S3 Fig The calibration curves of the prediction model which had the median AUC. (PDF)

S1 Table Details of hyperparameter tuning with optuna. (PDF)

S2 Table Main parameters of convolutional neural network. (PDF)

S1 File Materials and methods. (PDF)

## Abbreviations

18F-FDG-PET: Fluorodeoxyglucose Positron Emission Tomography
AI: Artificial Intelligence
AUC: Area Under the Curve
CI: Confidence Interval
CIRT: Carbon Ion Radiotherapy
CT: Computed Tomography
ECOG: Eastern Cooperative Oncology Group
Grad-CAM: Gradient-Weighted Class Activation Mapping
LC: Local Control
NSCLC: Non-Small Cell Lung Cancer
OS: Overall Survival
PFS: Progression-Free Survival
R: R (programming language)
RBE: Relative Biological Effectiveness
ROC: Receiver Operating Characteristic
SBRT: Stereotactic Body Radiotherapy
T stage (UICC, 8th edition): Tumor stage (Union for International Cancer Control, 8th Edition)
UICC: Union for International Cancer Control
XGBoost: Extreme Gradient Boosting.
