# Supplementary figures and images for: AI-based prediction of recurrence after carbon ion radiotherapy for early stage non-small cell lung cancer
Source: PLoS One. 2026 Feb 10;21(2):e0342481. doi: 10.1371/journal.pone.0342481 (PMC12890150; doi:10.1371/journal.pone.0342481)

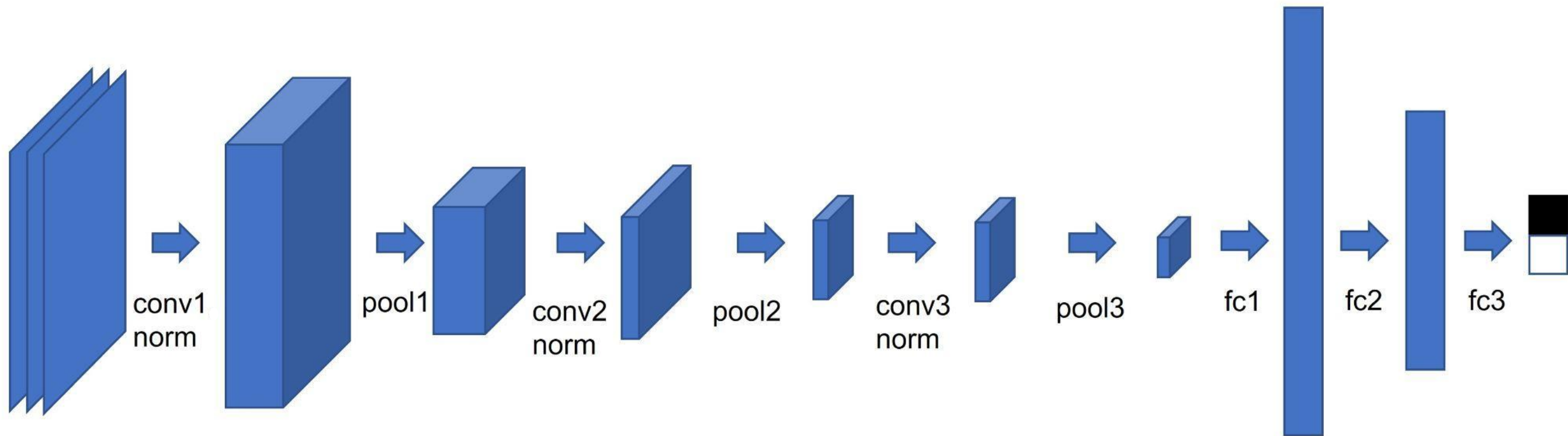

Supplement: S1 Fig — (PDF) [file pone.0342481.s001.pdf]

**A**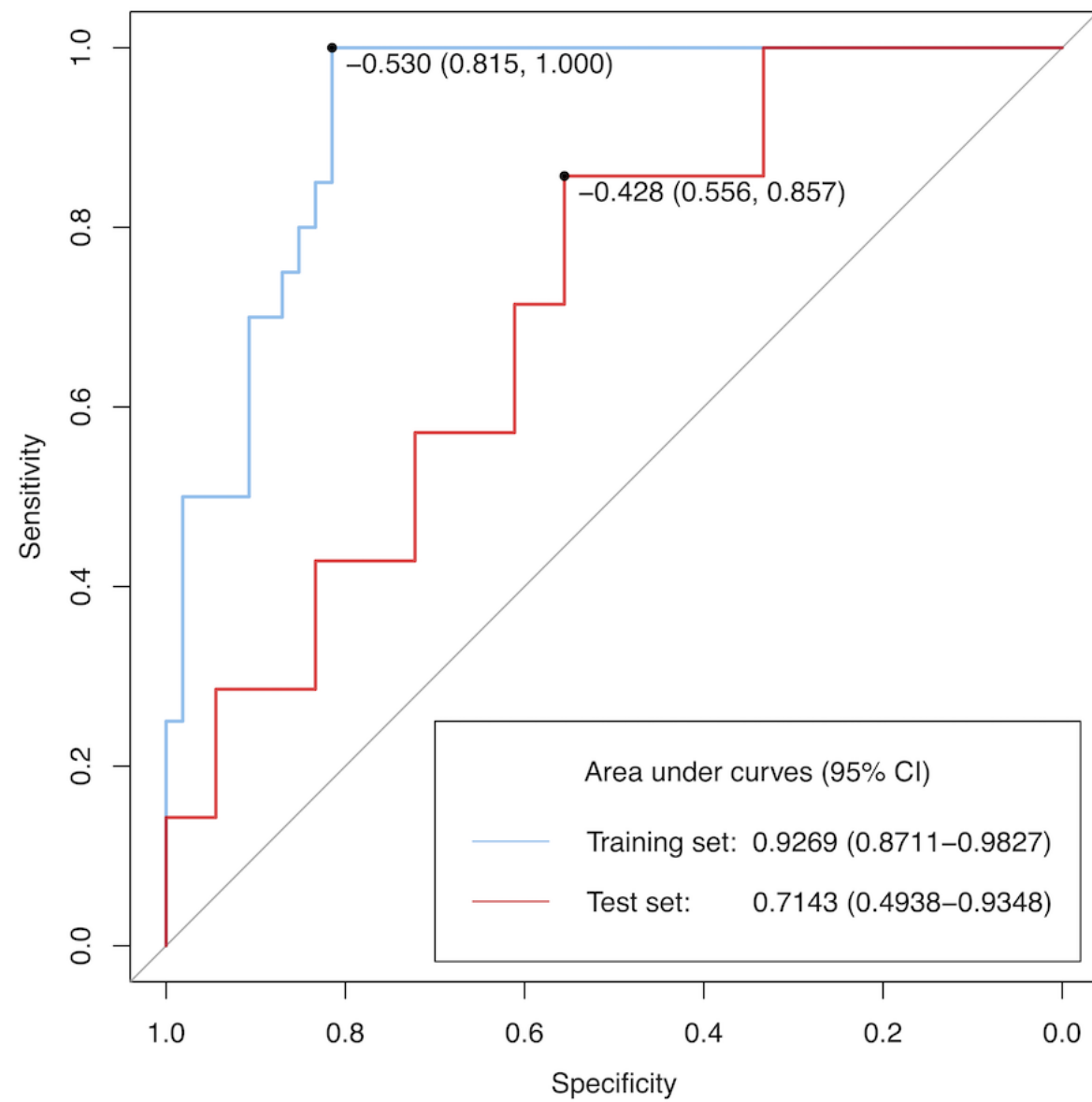**B**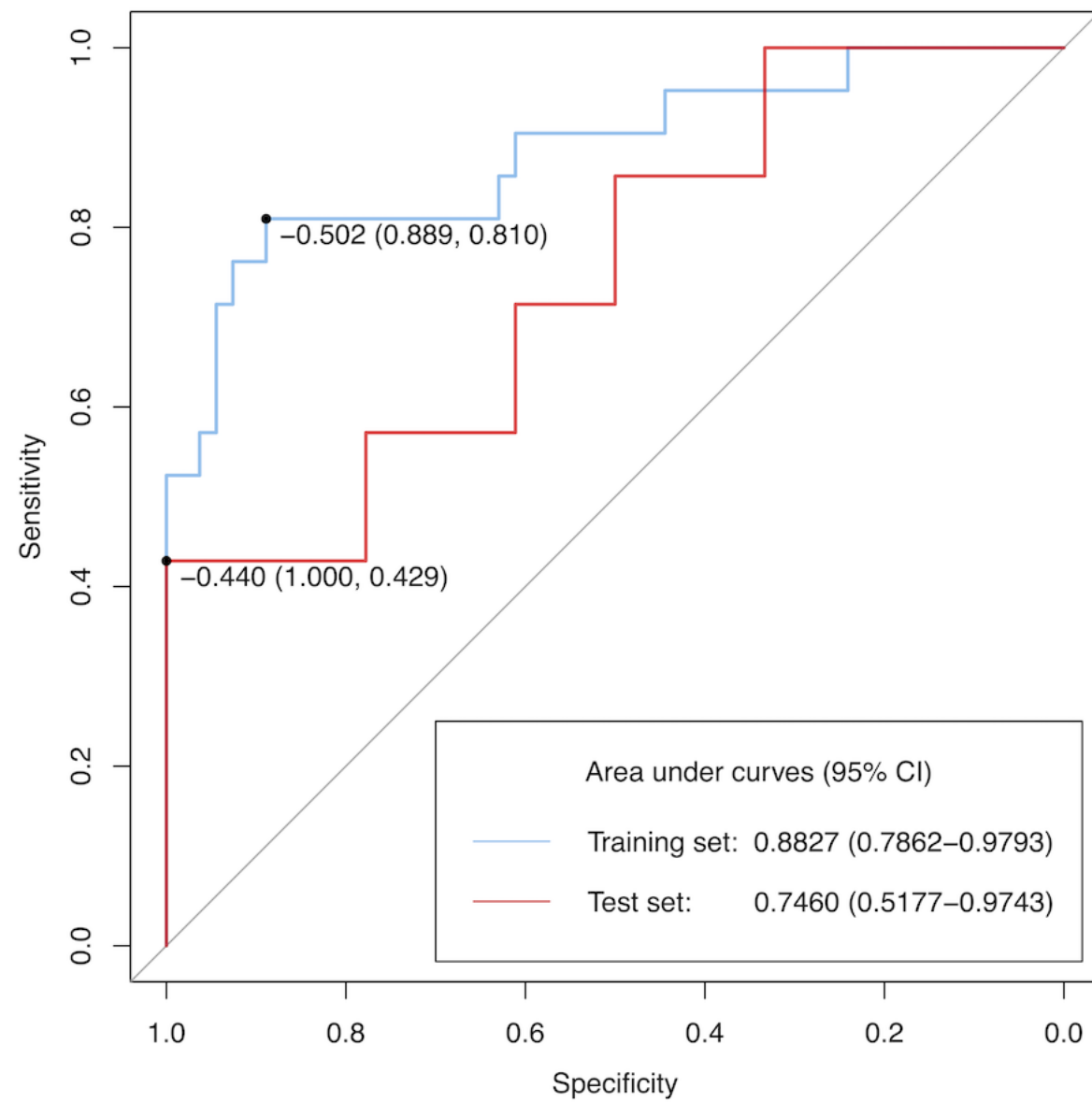**C**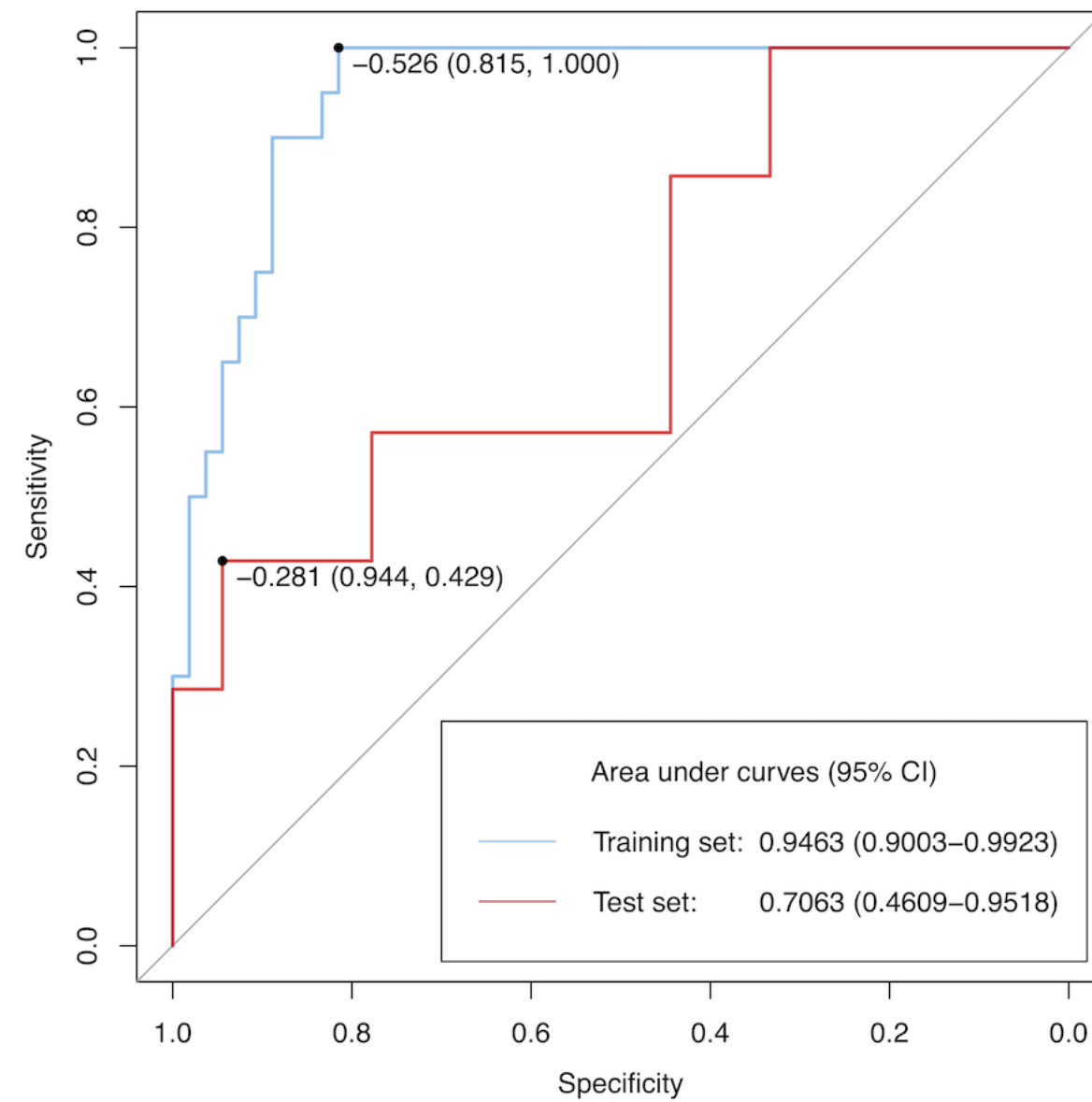

Supplement: S2 Fig — (PDF) [file pone.0342481.s002.pdf]

Calibration Plot

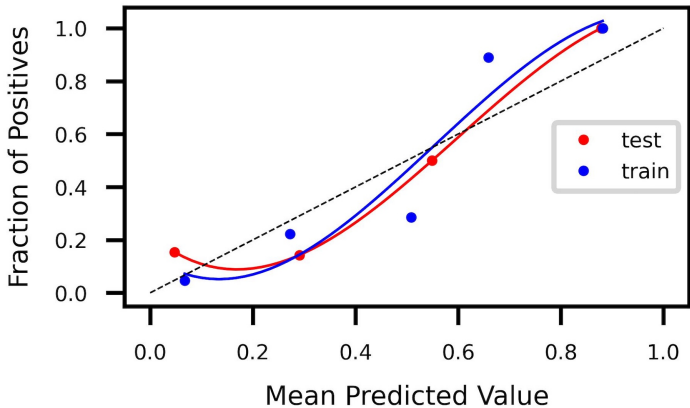

Supplement: S3 Fig — (PDF) [file pone.0342481.s003.pdf]
