# Supplementary material for: AI-based prediction of recurrence after carbon ion radiotherapy for early stage non-small cell lung cancer
Source: PLoS One. 2026 Feb 10;21(2):e0342481. doi: 10.1371/journal.pone.0342481 (PMC12890150; doi:10.1371/journal.pone.0342481)
Supplement: S1 Table — (PDF) [file pone.0342481.s004.pdf]

| Parameter | Search range | Optimized value |
|---|---|---|
| Learning rate | 0.01–0.3 | 0.012877826971672468 |
| Minimal weight of leaf node | 2–8 | 5 |
| Max depth of decision tree | 1–4 | 2 |
| Sampling ratio of explanatory variables | 0.2–1.0 | 0.996735636509729 |
| L1 Normalization term alpha | log(0.001)–log(0.1) | 0.07999287311006402 |
| L2 Normalization term lambda | log(0.001)–log(0.1) | 0.021035034483435338 |
| Normalization term gamma | log(0.0001)–log(0.1) | 0.00010983873699283094 |
