# Supplementary material for: AI-based prediction of recurrence after carbon ion radiotherapy for early stage non-small cell lung cancer
Source: PLoS One. 2026 Feb 10;21(2):e0342481. doi: 10.1371/journal.pone.0342481 (PMC12890150; doi:10.1371/journal.pone.0342481)
Supplement: S2 Table — (PDF) [file pone.0342481.s005.pdf]

Layer	Type	Input size	Parameter value	Output Size
Conv1	Convolutional	3×32×32	Kernel:(5, 5), Stride:(1, 1), Padding:(2, 2)	64×32×32
Pool1	Pooling	64×32×32	Maximum, Kernel:3, Stride:2,	64×14×15
Conv2	Convolutional	64×14×15	Kernel:(5, 5), Stride:(1, 1), Padding:(2, 2)	32×15×15
Pool2	Pooling	32×15×15	Maximum, Kernel:3, Stride:2,	32×7×7
Conv3	Convolutional	32×7×7	Kernel:(5, 5), Stride:(1, 1), Padding:(2, 2)	32×7×7
Pool3	Pooling	32×7×7	Maximum, Kernel:3, Stride: 2,	32×3×3
FC1	Fully Connected	32×3×3	-	500
FC2	Fully Connected	500	-	100
FC3	Fully Connected	100	-	2
