# Supplementary material for: AI-based prediction of recurrence after carbon ion radiotherapy for early stage non-small cell lung cancer
Source: PLoS One. 2026 Feb 10;21(2):e0342481. doi: 10.1371/journal.pone.0342481 (PMC12890150; doi:10.1371/journal.pone.0342481)
Supplement: S1 File — (PDF) [file pone.0342481.s006.pdf]

Materials and methods

Procedures of CIRT

The patient was immobilised using a thermoplastic shell (Shellfitter; Sanyo Polymer Industrial, Nara, Japan) and a pillow made of water-sclerogenic polymers (Moldcare; ALCARE, Tokyo, Japan). A simulated computed tomography (CT) image with 2 mm-thick slices was obtained after exhaling in two positions. A four-dimensional CT scan was also performed to assess respiratory motion and reconstruct four-dimensional images of each respiration phase. All the CT images were taken with Canon Aquilion LB CT Scanner (Canon Medical Systems, Tochigi, Japan).

The gross tumour volume (GTV) was delineated in the lung window. The clinical target volume (CTV) was generated by adding a 5 mm margin from the pulmonary parenchyma to the GTV. The inner margin was determined using four-dimensional CT with a 3 mm-setup margin. A margin, calculated as the square root of the sum of the squares of the inner and setup margins, was added to the CTV to create a planning target volume.

The clinical dose distribution was based on the physical dose multiplied by the relative biological effectiveness (RBE) of 3.0. XiO-N treatment-planning software (Elekta AB, Stockholm, Sweden; Mitsubishi Electric, Tokyo, Japan) was used to calculate the passive-scattering carbon-ion dose distribution.

The total prescribed dose was 52.8 Gy or 60.0 Gy (RBE) for the isocenter in four fractions over a week, with a fractional dose of 13.2 Gy or 15.0 Gy (RBE) at four treatment sessions per week. Respiratory-gated irradiation with a gating level of <30% of the wave height around the peak exhalation was applied at each treatment session.

Development environment

The experiments were conducted using a system running Python (version 3.9) and CUDA (version 11.6) for GPU acceleration, equipped with an NVIDIA GeForce RTX 3090.

The following Python libraries were used: NumPy (version 1.24), Pandas (version 1.5), tqdm (version 4.64), matplotlib (version 3.6), PyTorch (version 1.13), torchvision (version 0.14), xgboost (version 1.7), scikit-learn (version 1.1), and optuna (version 3.0).

Input CT images for CNN

The input data for the deep learning model were obtained from CT images. The central coordinates of the tumour were determined using calculations derived from DICOM-RT data. Two methods were used to calculate the central position of the tumour. The first method involved the direct utilisation of the isocentre position (DICOM Tag: (300a,012c)). The second method involved calculating the major axis of the tumour within each image slice. This was achieved by analysing

multiple closed contours, commonly referred to as “tumour contours,” obtained from the ROI Contour Sequence (DICOM tag: (3006, 0039)). The centre of the tumour was identified as the midpoint of the contour within the slice that displayed the longest major axis. Notably, an ROI Contour Sequence was consistently applied, and its association with a specific ROI name (private tag) was maintained. The selection between the two methods for determining the tumour centre was made following a visual assessment, favouring the method that provided a more accurate position.

The input data encompassed three squares, each extracted with its centre aligned with the central position of the tumour within the CT slice. In addition, two orthogonal planes were included. Alternatively, a cubic volume centred on the centre of the tumour was used. When three squares are used as inputs, each square is mapped to one of the RGB colour channels, resulting in the creation of an RGB two-dimensional image. To ensure size uniformity within the input data, isotropic resampling was employed to ensure consistent pixel spacing across the entire dataset.
